# Altruistic decision-making is associated with certain patterns of local brain functional connectivity

**DOI:** 10.1192/j.eurpsy.2023.1293

**Published:** 2023-07-19

**Authors:** D. Mitiureva, A. Kiselnikov, E. Terlichenko, V. Zubko, P. Kabanova, V. Abrosimova, E. Krivchenkova, D. Verkholaz, S. Skripkina, V. Udartseva, A. Komarova

**Affiliations:** 1 Lomonosov Moscow State University; 2 Institute of Higher Nervous Activity and Neurophysiology of RAS, Moscow, Russian Federation

## Abstract

**Introduction:**

The brain mechanisms of altruism cannot be strictly localized; therefore, the analysis of brain functional connectivity (FC) can reveal intrinsic mechanisms of altruism or, conversely, anti-social tendencies in behavior.

**Objectives:**

The objective was to investigate local FC patterns of altruistic decision-making using the “Pain versus Gain” (PvsG) paradigm.

**Methods:**

The sample included 38 participants (18 females), 21.2±2.1 y.o. who signed the informed consent form and filled in the Interpersonal Reactivity Index questionnaire (IRI). The study protocol was approved by the local ethical committee. The PvsG task consisted of the control (CC) and experimental condition (EC) with 20 trials, each with 6 possible decisions. In the CC, participants had to decide which finger the second fake participant (FP) to move (one of five fingers or no move). In the EC, they were given money (1000 rubles) and had to choose in every trial between self-benefit (to keep 50, 40, 30, 20, 10 or 0 rubles) or FP’s pain induced by the medical electromyostimulator (with 6 levels of intensity, from “highest” to absence), e.g., when a participant keeps no money, the FP receives no stimulation. The FP was not present, and his finger moves and hand reactions to the stimulation were pre-recorded and presented as feedback. 62-channel EEG was recorded simultaneously, and the time intervals for decision-making were used for the weighted Phase Lag Index (wPLI) computation between the reconstructed cortical sources. Spearman coefficients with p-values correction via permutations were calculated between FC difference (EC vs. CC) and the sum of money given out.

**Results:**

The money given out correlates positively with the Empathic Concern (R=0.38, p=0.01), Perspective-taking (R=0.42, p=0.01), Fantasy scale (R=0.4, p=0.01), and does not correlate with the Personal Distress scale (R=0.17, p=0.28) of the IRI. Significant correlations were found between the money given out and the FC between the right lingual gyrus (lg) and caudal ACC in 4-30 Hz band (R=0.54, p<0.001) and FC between the caudal ACC and left insula in 8-13 Hz band (R=0.58, p<0.001).

**Conclusions:**

The PvsG task is a valid paradigm for the investigation of brain mechanisms of altruistic decision-making. We described local FC correlates of prosociality formalized in the money given out which is associated with the measures of empathic concern and cognitive empathy. The ACC and insula are involved in salience network and pain matrix with the lg as an afferent, and their activity is modulated by empathy towards others. Thus, we claim that altruism depends on empathic motivation, which is associated with FC between these regions.

**Disclosure of Interest:**

None Declared

